# Beyond Climate Focus and Disciplinary Myopia. The Roles and Responsibilities of Hospitals and Healthcare Professionals

**DOI:** 10.3390/ijerph6031204

**Published:** 2009-03-19

**Authors:** John P. Ulhøi, Benedicte P. Ulhøi

**Affiliations:** 1 Centre for Organizational Renewal and Evolution, Aarhus School of Business, Aarhus University, Haslegaardsvej 10, DK-8210 Aarhus V, Denmark; 2 Dept. of Pathology, Aarhus University Hospital, Nørrebrogade 44, DK-8000 Aarhus C, Denmark; E-Mail: beneulho@rm.dk

**Keywords:** Sustainability, hospitals, roles, responsibilities, drivers and obstacles

## Abstract

This paper calls for the need to address climate change within the concept of sustainable development, in recognition of the interrelationships between environmental, economic and social systems. So far, health- providing organizations such as hospitals have paid surprisingly little attention to the relationships between environmental change (e.g. climate change) and human health, or between hospitals (as professional organizations) and their impact on sustainable development. Although it is usually such industries as the chemical, extractive and metal industries, etc., that are associated with environmentally harmful activities, there is also an urgent need to emphasize the roles and responsibilities of hospitals and their embeddedness in a wider ecological, economic and social context. The key objective here is to discuss the relevance of sustainability and environmental management issues in a sector that until now has conveniently ignored its roles and responsibilities in relation to sustainability issues. The paper concludes that arguments based on systems theory, environment, medicine, economics and innovation strongly urge hospitals to reconsider their present roles and environmental responsibilities.

## 1. Introduction

The general debate on the global environment has recently seen growing interest in the aspect of anthropogenic global warming, or the so-called enhanced greenhouse effect. However, while there is ample scientific evidence of the man-made effects on the environment [1], it is important not to limit the environmental debate in such a way as to divert attention from the critical roots of the problem and thereby focus more on abatement rather than avoidance strategies. Unsustainable development cannot be understood solely within the boundary of the natural sciences, however, but also needs to include economic, social, and cultural explanations. This paper argues that man-made climate change should be recognized for what it is – an important symptom, rather than the main problem, of increasingly unsustainable development. In consequence, we suggest repositioning the focus of the present environmental and climate debate within the context of sustainable development as defined by the World Commission for Environment and Development [2].

The concept of sustainable development, which was fiercely debated during the late 1980s and throughout the 1990s, refers to the capacity of ecological, economic and social systems to preserve vital processes, functions, productivity and diversity for future generations. However, surprisingly little attention has been paid to the relationships between environmental change (e.g. climate change) and human health, or between professional organizations such as hospitals and their roles and responsibilities in relation to sustainable development. This is highly unfortunate.

The aim of this paper is to shift the focus of attention of the sector towards sustainability and environmental concerns. The paper addresses some key challenges in relation to more sustainable health treatment in society. In so doing, we argue that part of the problem is due to the interdisciplinary and complex nature of the phenomenon of sustainability, which challenges the existing scientific assumptions and self-perceived roles and responsibilities of professionals in the health care sector.

The paper is organized as follows. The next section briefly defines the phenomenon of sustainability and discusses society’s main responses to increasing environmentally unsustainable development. Section 3 argues why hospitals have a key role and responsibility in reducing the environmentally detrimental effects of human activities. Section 4 discusses some of the challenges of the greening of the hospital sector. In closing, Section 5 summarizes the key reasons why, from a systems theory, environmental, medical, economic and innovation point of view, hospitals should undertake new roles and more responsibility in relation to environmental issues.

## 2. Sustainability and Society’s Response to Increasing Environmentally Unsustainable Development

Sustainability is a concept which links three systems and related activities, processes and mechanisms: (i) ecological systems, (ii) economic systems, and (iii) social systems. All three systems need to be considered when planning avoidance strategies and assessing the potential impact of new initiatives and/or developments. This development concept, which has become widely adopted by policymakers since its introduction by the World Commission on Environment and Development Commission in 1987, represents development without harm, i.e. ”...development that meets the needs of the present without compromising the ability of future generations to meet their own needs” [2]. The WCED report recognizes that environmental problems and their solutions should not be addressed in isolation from economic and social systems, including related cultural and ethical aspects.

The concept of sustainable development also recognizes that imbalances in one (sub)system are likely to affect and/or be affected by other (sub)systems, e.g. poverty, war and/or unregulated economic greed, which may result in little or no consideration for surrounding ecological systems, natural resources and/or carrying capacity. Thus, the wider negative consequences of wars and poverty (in terms of loss or deprivation of lives and ecological resources) tend to be exacerbated by the ignorance of such linkages. Moreover, sustainable development as introduced by The World Commission on Environment and Development also implies that, from a moral point of view, existing generations should not be allowed to deprive future generations of their access to similar vital natural resources. The 1987 WCED Report led to a variety of initiatives in the form of voluntary European and/or international environmental standards and/or systems, including The British Standard 7750 (BS7750) (http://www.quality.co.uk/bs7750.htm), The European Eco-Management and Auditing Scheme (http://www.iema.net/ems/emas) (EMAS), and the international ISO (http://www.iso.org/iso/iso_catalogue/management_standards/iso_9000_iso_14000/iso_14000_essentials.htm) standards.

Several high-profile international meetings (The Montreal Protocol on Substances That Deplete the Ozone Layer came into force on January 1989 in Helsinki; The Kyoto Protocol at the UN Conference on Environment and Development, held in Rio de Janeiro, Brazil, June 3–14, 1992; The Asia-Pacific Partnership on Clean Development and Climate, in Sydney, January 2006) have been held during the past couple of decades, the aims of which have been to reduce and/or regulate some of the environmentally detrimental impacts of society’s productive and reproductive activities. This development has paved the way to the introduction of integrated environmental management systems which subsequently has led to an ongoing upgrading of existing organizations’ management systems, designed to ensure the efficiency of administrative ’housekeeping’ systems. However, recent findings suggest that hospital management is slow to adopt modern management techniques designed to maximize operational and strategic improvements [3]. Studies of re-engineering work processes in US hospitals suggest that successful implementation requires appropriate facilitation [4]. Recent studies of US hospitals that have adopted case management approaches conclude that institutional influences aimed at achieving or maintaining legitimacy may be as strong a motivation incentive as economy [5].

Two main approaches to environmental protection have been widely embraced: (i) permissions and standards (direct regulation of behaviour) and (ii) environmental taxation (indirect regulation of behaviour), where the latter is normally perceived as an economic approach [6]. The latter approach involves economic incentives to innovate in order to prevent unnecessary environmental costs and giving manufacturers a choice when faced with environmental problems. Since the early 1990s, however, there has been an increasing focus on self-regulative approaches, i.e. approaches based on voluntarism on behalf of the polluter (the manufacturer).

Increasing environmental unsustainability in terms of climate change, ozone depletion, pollution of the atmosphere and oceans, uncontrollable urbanization, the escalating loss of biological diversity and shortage of fresh drinking water, however, poses a major challenge to public health, and has been addressed in several of UNEP’s Global Environment Outlook assessments (http://www.unep.org/geo/) over the years.

## 3. Sustainability and Its Relevance to Hospitals

More than a century ago, (disease) treatment and care and (disease) prevention and control began developing into two interrelated, albeit increasingly separate, fields [7]. Where the former took the path of in-depth specialized knowledge, for treating and curing pathological and/or dysfunctional conditions in individuals, the latter took a more interdisciplinary and broad (also involving the social sciences) approach (involving micro and macro perspectives) to control and prevent diseases from spreading, or getting out of control, among populations of individuals.

Today, hospitals are generally regarded as organizations whose main mission is to help human beings in need of surgery and/or medical assistance, as well as to advise people on healthy ways of living. This implies that hospitals should find it difficult to justify behaviour that may threaten human health and/or contribute to the growing depletion of natural resources and/or continuing inefficient energy and resource management. Hospitals are also increasingly likely to be affected by harmful environmental change in terms of loss of lives and a rising number of (new) diseases. Evidence has shown that changes and variations in climate affect health outcomes [8,9]. Figures from the past three decades indicate that the negative environmental impact on health can be counted in the tens of thousands lives [10].

There are other relevant sustainability-hospital interactions that invite for re-considering the roles and responsibilities of hospitals in relation to environmental sustainability: (Public) hospitals are typically large employers and spenders of public resources. Their activities and buildings affect the environment through their use of energy and resources, including non-renewable ones [11], as well as waste production and handling, and they are toxic waste producers [12], leading to problematic waste handling problems. Hospitals often dominate the local environment in terms of employment, and thus generate a lot of traffic.

Hospitals have to live up to expectations that transcend purely economic or efficiency criteria, since they are established precisely to save, treat and/or heal human life. Unfortunately, demand for rescue and/or acute treatment is neither stable over the year nor equally distributed throughout the day. It thus follows that designing the productive capacity of a public hospital requires a high degree of flexibility and buffer capacity. Moreover, health care professionals typically have values (institutionalized as well as legitimized) that transcend pure economic goals and self-maximizing behaviour, e.g. always trying to save life whenever possible. Hospitals are organizations staffed with highly specialized professionals with little or no tradition for subscribing to holistic and/or preventive perspectives. Last but not least, health is by definition a sustainable state [13].

The recent emergence of various NGOs and/or interorganizational initiatives, such as Healthcare Without Harm (noHarm) (www.noharm.org), Green Guide for Healthcare (www.gghc.org) (GGHC), Hospitals for a Healthy Environment (www.h2e-online.org) (H2E), and the Collaborative on Health and the Environment (http://www.healthandenvironment.org), suggest that the healthcare sector has at last started to recognize some of its roles and responsibilities. In January 2009, The National Health Service’s Sustainable Development Unit (http://www.sdu.nhs.uk/) in England under the auspices of the Office of the Strategic Health Authorities has launched a Carbon Reduction Strategy, laying down the scale of reduction in carbon required for the National Health Services to progress towards the Climate Change Act requirements and suggests key actions for becoming a leading sustainable and low carbon organization. Much more can be achieved by actively implementing environmental management practices and cleaner technologies if sustainability is integrated into the value systems of healthcare professionals. Another initiative worth considering is to integrate environment-related elements into existing healthcare-related education systems. Earlier studies from industry and related providers of education have emphasized the critical role of such education in relation to sustainability [14].

## 4. Discussion

There is overwhelming scientific agreement regarding the relationship between the emission of greenhouse gasses and climate change (The Intergovernmental Committee on Climate Change [1]). Furthermore, there seems to be increasing recognition that climate change and human health are strongly interrelated [8]. Reframing the present environmental and climate debate within the concept of sustainable development implies a greater focus on some of the problematic drivers of the present unsustainable socioeconomic system. From the point of view of sustainable development, it seems unlikely that the planet can grow out of existing environment- and sustainability-related challenges. Universally unlimited and uncontrolled material growth is incompatible with a global sustainable future. Rather, it seems that difficult political priorities and regulations (local and international), followed by re-evaluations of existing consumption patterns and value preferences, are needed.

### 4.1. Obvious Reasons for Undertaking Increased Sustainability Related Responsibilities in the Hospital Sector

There are some obvious and direct reasons why unsustainable development leading to climate change is of core relevance to hospitals. Firstly, climate change can have damaging effects on human health, thus threatening the core mission and services of hospitals (proving and securing health service). Secondly, by actively contributing to a sustainable development but reducing energy and resource consumption can lead to direct economic benefits because such changes translate into economic savings for hospital management – savings that can then be used to improve the core services provided by hospitals. Given that the present unsustainable development is affected by human choice, it is worth noting that a single change in behaviour, such as the replacement of high energy-consuming technologies with low energy-consuming attitudes can have significant environmental benefits. However, other environmentally beneficial changes may require an ongoing change in human and social behaviour, e.g. sorting and collecting recyclables [15]. While the former normally requires little effort to be successfully implemented, the latter requires a long-lasting change of behaviour, and with it increasingly higher investment and risk of failure. Thirdly, by embarking on the social responsibility related to a sustainable development, hospitals can demonstrate that they are taking their corporate social and environmental aspects related to sustainable practices seriously while at the same time improving their general reputation as well as their relationships with their external stakeholders.

### 4.2. Barriers towards Increased Sustainability Related Responsibilities

These obvious advantages are however up against important inertia steaming from how our society and its economic development perceive success. The evolution of socioeconomic systems unfortunately seems to be rooted in one-dimensional criteria of success (growth) and nested within a fundamental belief that all human beings are exclusively opportunistic and self-maximizing agents. Mainstream economic theory has difficulties in coming to terms with the fact that economic systems cannot produce value in splendid isolation from the environment’s ability to absorb the effects of such production. There is a need for economic theory to include nature’s carrying capacity not as a good that can be traded on the market, but as a precondition for future socioeconomic activity. The challenge here is how to ensure material prosperity in the present without leaving future generations worse off. Other factors also need to be considered, including the qualitative aspects of life and development and intrinsic environmental values (e.g. biodiversity and aesthetical properties). Moreover, human behaviour is influenced by highly institutionalized values and attitudes as well as its consequences and existing routinized behaviour. It can therefore be expected that a lasting change of behaviour will require attitudinal, educational and/or training initiatives.

Although mainstream economic theory portrays human decision-makers as self-optimizing, rational creatures, not all decision-makers are entirely selfish or rational. If this were not the case, there would be no bottom-up charity organizations or non-contractual and/or trust-based networks. Put another way, some people have an unselfish concern for the wellbeing of other people. Health professionals, for example, are trained to help other people in need of surgery and/or medical assistance. Such characteristics may also favour an increased willingness to care for the environment, a behavioural property normally referred to as altruism. Such ’actively caring behaviour’ has been described as a mediating factor between environmentally responsible behaviour and personality characteristics related to self-affirmation [15]. Empirical tests have supported this [16].

### 4.3. Complex Systems Theory

Apart from the unintended adverse effects of society’s productive functions, the way in which science in Western economies has been organized and practiced is also a potential barrier to conceptualizing and responding to environmental problems and their origins. Mainstream scientific inquiry in the West is based on the assumption that systems can be decomposed into their parts, that system components can be identified, separated, decomposed and categorized, that they can be subject to simplified and independent analyses, and that the sum of the parts is equal to the sum of the whole. Such a reductionist approach has led to a growing decompartmentalization of sciences into increasingly smaller and more specialized subfields. In many cases, however, this way of organizing the production of scientific knowledge has worked very well. Natural and technical sciences, however, tend to be rooted in a ’man-over-nature’ belief, as though we as a species can transcend our environmental dependency and free humanity from the constraint of the carrying capacity of the biosphere [17]. However, in the case of dynamics, complexity and interlinkages of systems and subsystems – e.g. problems related to unsustainable developmental trajectories – this requires approaches that recognize interlinkages between the interacting systems.

Circularity of action between parts of a dynamic system results in feedback [18]. Feedback mechanisms may accelerate or enhance the output created by a stimulus that has already been activated (positive feedback), and other mechanisms may reduce the output back to its normal range of functioning (negative feedback). In cases of low-to-moderate variation in terms of systems, dimensions, levels, scales (i.e. low complexity), etc., the mainstream scientific approach has been very successful. Where there is a lot of variety in levels, systems, dimensions and scales (high complexity), however, this approach cannot stand alone but needs to be supplemented by a different scientific approach. An interdisciplinary, complex systems science has emerged to meet the need to explain and/or predict complex systems behaviour. Translated into a social system perspective, existing theory can account for the way in which systems and complex entities are capable of maintaining an independent existence - albeit not totally separate from the environment. These are situations where structural changes and overlap with other systems can take place without the disappearance of individual identity or severance from a niche [19]. Such systems are called viable systems [20].

A complex systems theory perspective recognizes that systems – natural as well as social – are influenced by complex subsystem interrelationships and dynamic feedback mechanisms, i.e. how temporary and unintended system-level order emerges spontaneously from actors, repeated activities, and interactions of lower-level system constituents without the intervention of hierarchical coordination and/or control [21]. Interactions between system components and the resulting feedback mechanisms remain a key focus of complexity studies. Rather than being predominantly causal, complexity theory accepts that indiscriminate incidents may also occur. We propose that the phenomenon of sustainability qualifies as a complex phenomenon. Taken as a science, sustainability and environmental processes transgress traditional disciplinary boundaries. While we are not saying that a complex systems approach can replace existing and valuable theories within our own and/or other related disciplines, we do suggest that such an approach may be worth considering as a supplementary approach to conceptualizing the relationship between hospitals and sustainability, including hospital administration.

Notwithstanding, a lot of uncertainty surrounds existing estimates of environmental effects. Modelling and forecasting are not so much exact sciences as a helpful means, based on scientific standards, to produce *indicative* rather than *predictive* results. The complexity and interrelatedness of the human-environment equation makes attributing specific causes and effects far from straightforward. Moreover, many effects are difficult to quantify, and are not necessarily manifested immediately, e.g. the socioeconomic and sociocultural changes of adverse environmental effects (climate change, resource depletion). In our empirical studies, we may often be tempted to focus on proximal factors in immediately recognizable pathological conditions. Distant or complex causal mechanisms related to systems interactions not well understood, or immediately recognizable or easily accessed in terms of data availability and/or quality, tend to escape our attention in clinical and empirical field studies. Moreover, unintended and/or unwanted potentially detrimental factors do not automatically show up in our data set devoid of theoretical considerations – they are influenced by the way we think a priori about the problems under consideration, i.e. by the choice of framework that guides our selection and/or omission of key variables [22].

The value chain of medical treatment, from diagnosis of partial malfunctioning to post-treatment follow-up services, is seldom considered in its totality. In a hospital, for example, individual parts of the overall system have difficulty in appreciating lower level actions (or the lack thereof) and their potential effects on larger parts of the system (and vice versa). Hospitals are what in organization science has been characterized as professional bureaucracies. This type of organization is interesting from the point of view of control and coordination. Control resides among the professionals providing the services [23]. Thus, the ability of upper echelon managers to influence the behaviour of such professionals is somewhat constrained. Contrary to traditional hierarchies, coordination in professional bureaucracies relies to a large degree on peer and collegial processes and mechanisms influenced by norms that are not always reconcilable with economic efficiency [24]. However, as pointed out by recent research [25], intervention can take many forms, ranging from primarily environmental, through educational and organizational, to technological in nature.

## 5. Implications

In undertaking new roles and/or increasing responsibility for ongoing environmental degradation, the hospital sector and its education system will face some important challenges. At the operative level, hospital staff will have to understand and accept that they are co-responsible, that they can make a difference by changing their behaviour, and that high environmental standards are fully compatible with high medical standards. The educational system is one institution which affects values and professional roles and responsibilities. This paper implies that existing education in the healthcare sector fails to give adequate attention to the roles of the larger eco-systems within which it operates. In order to support the ongoing change of values and preferences that are incompatible with high environmental standards, the related educational system must fully recognize these roles. At the strategic and/or administrative level, hospital management needs to adopt the most modern and externally certifiable environmental management systems (e.g. ISO14001), and be compared not only intrasectionally (with other leading hospitals) but also intersectionally, i.e. against the highest standards implemented by other leading business organizations. Accepting more responsibilities can also lead to new roles in greening other ’unusual suspects’ in the public and private service sector. Adding the wider implications of sustainability, (public) hospital managers also need to expand their cost and benefit analyses to include the buying of materials and/or products. Focusing solely on price may lead to unintended effects, such as erosion of the local economy, which in turn leads to increasing unemployment and social and health problems.

A key implication of our discussion is that the present international and local environmental debate should take place within the concept of sustainable development. This implies a recognition that solving serious problems such as climate change will require not only addressing the treatment perspective (e.g. reduction of CO_2_), but also the more long-term prevention perspective (change in behaviour and technology). More research on how emerging cleaner technologies, ITs and nano technologies can contribute to solving some of the problems is needed. Medical technology entrepreneurs and public policy-makers in the field also need to consider changes in privacy protection and knowledge-sharing following from increased cross- and interdisciplinary collaboration, and the need for new innovation and business models and risk-tolerant finance.

A re-evaluation of roles and responsibilities in the healthcare sector suggests closer ties and collaboration between medicine and epidemiology [7]. With regard to sustainability and some of its important related issues (climate change), there is a need to include sciences other than natural sciences in future efforts. Such interdisciplinary collaboration in the production of new scientific insights is very rare, and presents serious challenges in terms of cross-disciplinary communication and tolerance.

History has taught us that sustainability is hard to achieve if left to unrestricted market forces. The inherent nature of unregulated market mechanisms, i.e. its underlying value system (quantitative and economic growth) and time horizon (short term) is incompatible with the preservation of essential environmental resources and capabilities. Unsustainable values and time horizons need to be replaced by sustainable ones through ongoing efforts via the educational system and workplaces, where enthusiasts and innovators may act as exemplars. For this to happen more smoothly within existing time and resource constraints, hospitals need to engage in partnerships rather than work alone on this challenge, and new tools are required to ensure that intersectional action becomes integrated in hospitals’ present modus operandi [26].

## 6. Conclusions

If we as scientific investigators are to avoid blindly groping around in the dark, we need to step back for a better look while at the same time exchanging insights across the different sciences. Not daring to grasp the opportunity of interdisciplinary dialogue will leave us in our highly specialized but isolated subfields, more occupied with fine-tuning the relative fit between individual pieces of the larger jigsaw. Few if any will thus be in a position to see and interpret the wider picture.

There are theoretical, ecological, medical, economic, ethical and innovation-related reasons to undertake more environmental responsibilities. Systems are interlinked, and over-exploitation and/or imbalances in one system affect other systems and subsystems, with potentially non-linear and accelerating changes (the systems theory argument). Human activity, such as health treatment, is a potential threat to the natural balances of the ecological systems (the ecological argument). Hospitals have multiple negative environmental effects that threaten human health and wellbeing (the medical argument). Operating and managing hospitals involves high energy and other resource consumption, and the inefficient use of such energy and resources will translate into higher costs than necessary (the economic argument).

Operating and managing hospitals in environmentally unsustainable ways will have a direct and indirect negative effect on human lives and well-being, and is thus incompatible with the raison d’être of such organizations, i.e. the treatment and curing of human diseases (the moral argument). Last but not least, as late starters and heavy producers of direct and indirect negative environmental effects, hospitals have unrealized opportunities for assuming new roles and responsibilities (as an active guard against life-threatening and unsustainable developments). Moreover, as knowledge-intensive organizations, they have the intellectual potential to come up with innovative solutions to existing unsustainable behaviour (the innovation-related reason).
